# Single-Cell States in the Estrogen Response of Breast Cancer Cell Lines

**DOI:** 10.1371/journal.pone.0088485

**Published:** 2014-02-25

**Authors:** Francesco Paolo Casale, Giorgio Giurato, Giovanni Nassa, Jonathan W. Armond, Chris J. Oates, Davide Corá, Andrea Gamba, Sach Mukherjee, Alessandro Weisz, Mario Nicodemi

**Affiliations:** 1 European Molecular Biology Laboratory, European Bioinformatics Institute, Hinxton, Cambridge, United Kingdom; 2 Department of Medicine and Surgery, Laboratory of Molecular Medicine and Genomics, University of Salerno, Baronissi, Salerno, Italy; 3 Centre for Complexity Science, University of Warwick, Coventry, United Kingdom; 4 Institute for Cancer Research and Treatment (IRCC), University of Turin School of Medicine, Candiolo, Turin, Italy; 5 Department of Applied Science and Technology, University of Turin, Istituto Nazionale di Fisica Nucleare (INFN), Human Genetics Foundation (HuGeF), Torino, Italy; 6 Netherlands Cancer Institute, Amsterdam, The Netherlands; 7 Department of Physics, University of Naples Federico II, CNR-Spin, Istituto Nazionale di Fisica Nucleare (INFN), Napoli, Italy; ENEA, Italy

## Abstract

Estrogen responsive breast cancer cell lines have been extensively studied to characterize transcriptional patterns in hormone-responsive tumors. Nevertheless, due to current technological limitations, genome-wide studies have typically been limited to population averaged data. Here we obtain, for the first time, a characterization at the single-cell level of the states and expression signatures of a hormone-starved MCF-7 cell system responding to estrogen. To do so, we employ a recently proposed model that allows for dissecting single-cell states from time-course microarray data. We show that within 32 hours following stimulation, MCF-7 cells traverse, most likely, six states, with a faster early response followed by a progressive deceleration. We also derive the genome-wide transcriptional profiles of such single-cell states and their functional characterization. Our results support a scenario where estrogen promotes cell cycle progression by controlling multiple, sequential regulatory steps, whose single-cell events are here identified.

## Introduction

Cellular responses to estrogens are characterized by a transcriptional activation and/or repression of specific subsets of genes, whose characterization will provide essential information on the molecular and genomic pathways of the hormone-responsive breast cancer (BC) phenotype. To this aim, estrogen responsive BC cell lines are useful model systems because of their deep transcriptional similarities with ER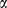-expressing breast tumors [1], [2]. Their response to estrogens has, thus, been deeply studied to try to characterize the structure of the process, and many advancements have been made. Nevertheless, a genome-wide quantitative analysis of the system at the single cell level is still lacking. This is related to an intrinsic limitation of current major time course genome-wide assays. In fact, time course data based on technologies such as microarray and RNA-seq can only capture population averaged expression levels. Yet, even if cells have been perfectly synchronized at the initial time point of the time-course, they will rapidly become a heterogeneous mixture because of the intrinsic stochasticity of cell state transitions. As a result, while such high-throughput techniques allow for a genome-wide characterization of the transformation of the population, they do not directly provide information on the cell states and expression signatures at the single-cell level.

To circumvent the above problems, we employ a quantitative analysis method capable to exploit population average data, e.g., microarray, and to dissect the single-cell events involved in the process. The method was previously used to investigate reprogramming of mouse embryonic fibroblasts into induced pluripotent stem cells over four weeks [3]. Here we consider a different biological system, a BC model, characterized by a much shorter time scale, 32 hours.

In our approach, the dynamics of a single-cell is described, via a Markov model, as a sequence of transitions between a network of different single-cell states. In this way, the cell distribution over the states and the population averaged, genome wide transcriptional levels can be derived in terms of the single-cell state transcriptional profiles and the transition rates across the states. Conversely, by fitting the population data, e.g., microarray data, the single cell states and transition rates can be obtained, thus providing a description of the system at a single-cell level.

More precisely, in the approach used here, the single-cell dynamics is described by a continuous time/discrete state Markov model. Coupling this approach with the use of advanced statistical methods and subsequent statistical analysis, we can determine, for the first time in a quantitative manner: *i)* the most likely number of single-cell states occurring in the BC estrogen-response process; *ii)* the transcriptional profiles of such single-cell states and their marker genes; *iii)* the key functional activities occurring in each single-cell state; and *iv)* the cell residence times and transition rates across the network of states. Here, in particular, we investigate the response to estrogen of a breast cancer MCF-7 cell model. We consider one of the largest available microarray time-course dataset of a MCF-7 hormone-starved system exposed to estrogen along 32 hours [4].

### Cell Systems and Datasets

The system considered here has been developed by Cicatiello et al. [4] who reported an extensive microarray dataset consisting in the time-course expression profiling of hormone-starved MCF-7 and ZR-75.1 model cells exposed to estrogen across 32 hours. The microarray data, including 12 time points, were extracted for 4960 noise-filtered genes, differentially expressed during the time-course assay [4]. In particular, a subset of 1270 genes has been shown to share a similar transcriptional response to estrogen in the two cell lines as described in Ref. [4]. They are referred to as common “estrogen-regulated” (E2R) genes. Cicatiello et al. [4] also performed ChIP-seq experiments to identify primary targets of ER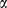 which led to the identification of 218 primary target genes (below named “primary genes”), i.e., E2R genes having an ER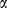 binding site within 10 kb from the transcription starting site (TSS). Finally, by matching the target sequences of transcription factors encoded by the primary genes with the sequences of E2R genes, 11 genes encoding transcription factors (“primary TF genes”) that affected expression levels of downstream genes were identified in that study.

## The Model

**Figure 1:**
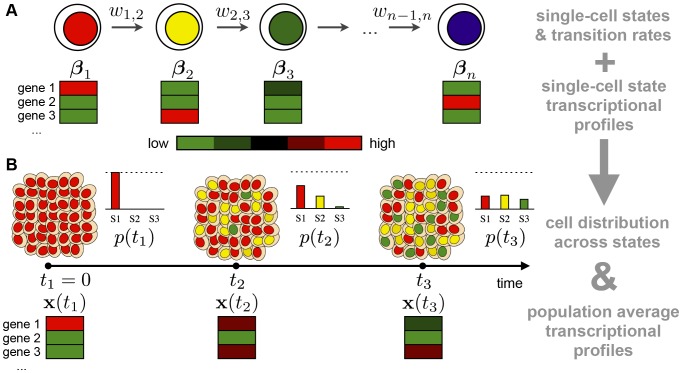
A schematic overview of the Markov model. (**A**) The dynamics of a single-cell is modeled as a sequence of transitions between 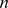 different cell states, via a Markov model, following [3]. The transition rate of a cell from state 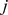 to state 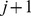 is named 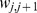, and the single-cell gene expression levels in state 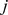 are named 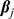 (i.e., 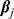 is a vector with the state transcriptional profile): in the example, genes in red (green) are up-regulated (down-regulated) in the corresponding state. (**B**) Population properties. An initially homogeneous cell population becomes heterogeneous because of the intrinsic stochasticity of cells which distribute over the different states. Microarray time course data record the average expression of genes at time 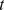, 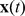. The Markov model (panel a) can connect the single-cell behavior to the population behavior. As illustrated in the rightmost column of the figure, by use of the single-cell transition rates, 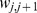, and state transcriptional profiles, 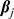, the time-dependent cell distribution across the states, 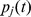, and the predicted average transcriptional profile, 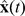, can be derived. Conversely, by fitting the microarray data, 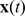, with the model predictions, 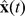, the properties of the single-cell states (the 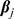
′s) and transition rates (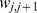
′s) can be extracted. Note that, in general, the average expressions, 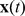, are different from the state profiles, 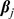, as seen in the example.

In the approach considered here [3], we assume that upon activation a cell visits a sequence of 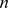 states (**Fig. 1A**). We outline here the method considered and refer to **Text S1** for further details. In our model the transitions between the single cell states are stochastic and described by a continuous-time Markov process. Although more complex cases can be considered (see [3] for technical details and discussion), for simplicity we focus here on linear state networks, with 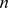 states. The dynamics of a cell is defined by the transition rates, 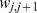, between all pairs of consecutive states 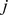 and 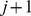, and the single-cell states by their expression signatures: we indicate with 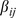 the expression of gene 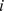 in state 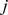. In our notation we name 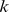 the total number of genes, i.e., 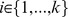. To be precise, since gene expressions still fluctuates in single cells, our state expressions refer to a single-cell average level [5], [6]. In the following we also use the symbol, 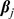 to represent the vector of the gene expressions for state 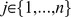 (**1A**).

**Fig. 1B** illustrates an example of the changes occurring in a cell population which results from the transition at the single cell level: while all cells are synchronized in state 1 at 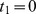, at 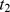 we see that about 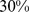 of cells have transited to state 2, i.e., the probability to find a cell in state 2 is 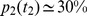, while a few cells have already reached the third state. At 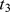 the population is highly heterogeneous with about 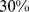 of cells being in each of state 1, 2 and 3 (i.e., 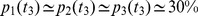). A microarray measure in such a heterogeneous population will capture the average expression of the genes, 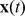, across the single-cell states which have been populated. Yet, the crucial point is that the average expression, 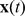, in general does not represent faithfully the single-cell state profiles. For instance, as illustrated in the example of **Fig. 1B**, gene 1 is highly expressed in state 1 while its expression drops sharply in the transition to state 2. However, the measure of its expression on the heterogeneous population is still comparatively high at time 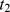 so that information is lost in the mixture of cell states. As we explain below, our method of analysis allows addressing these issues and dissecting the dynamics at a single-cell level.

The transition rates, 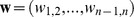 define univocally the dynamics of the cell population via the master equation of the Markov process:
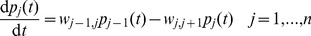
(1)


Here 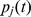 indicates the fraction of cells in state 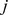 at time 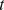 (and, in our notation, 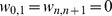). The master equation can be exactly integrated and the analytic form of 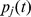 can be obtained as a function of the rates 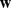 (**Text S1**). The parameters, 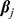, of the state expression profiles are “static” (i.e., they do not change over time) representing the features of the fixed single-cell states. The entire set of the model parameters is indicated below with 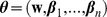.

The population-averaged expression 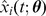 of gene 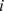 at time 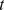 predicted by the model is, thus, given by:
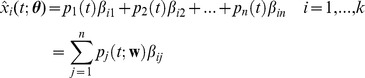
(2)where in the last line we have explicitly shown the dependence of the probabilities 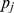 on the transition rates.

In general, in a linear network of 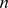 states (**Fig. 1A**), the model parameters are the 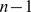 transition rates, 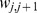, and 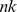 state-specific expression levels of the genes, 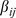. Suppose we have the expression level of 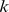 genes across 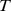 time points (in our notation 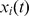 is the measured expression of gene 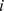 at time 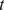): if the number of the model unknown parameters, 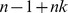, is less than the number of data, 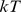, we can fit the data and derive the single-cell parameters. More precisely, we can infer the model parameters, 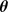, by fitting the measured gene expressions, 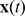, with the model predictions, 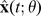: 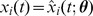 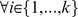 and 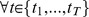. The model fitting algorithm used in our study is summarised in the Methods section and discussed in greater detail in **Text S1**.

## Methods

### Data

We considered the microarray dataset from Cicatiello et al. [4], deposited in the ArrayExpress database (http://www.ebi.ac.uk/microarray-as/ae/) with accession numbers E-TABM-742 and E-MTAB-131, consisting in time-course expression profiling of hormone-starved MCF-7 and ZR-75.1 exposed to estrogen across 32 hours. RNA was extracted before stimulation (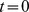) and at 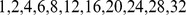 hours of exposition. Data were preprocessed as in Cicatiello et al. [4], where the procedure is described in details. In brief, data were normalized with Quantile [7] normalization and only genes having a detection p-value 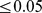 in at least one time point were considered. To identify differentially expressed genes we used the ILLUMINA DiffScore. The selected genes have DiffScore 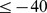 or 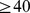, corresponding to a p-value of 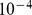, in at least one time point with respect to the control (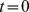). This procedure led to identify 4960 and 4106 noise-filtered genes responding to estrogen respectively in MCF-7 and ZR-75.1 cell lines.

We focus on transcription data as they are currently available, but our method can be applied to consider other time-varying data, such as epigenetic data, or important information on chromatin organization [8]–[10], to directly identify state-specific epigenetic signatures and spatial conformations along with expression patterns (to this aim other specific models can be associated to the present one [11], [12], along with other state-defining properties [13]).

### Model Fitting

As the model is characterized by a large set of parameters, a least-squares fit can be inadequate to reliably estimate its parameters, 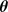 [14]. Thus, we employed a maximum-a-posteriori approach (MAP), where available prior knowledge is used to regularize the estimation. The MAP estimate is obtained by maximizing the posterior density 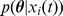 (i.e., the probability density over parameters 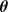 given the observed data) with respect to the parameters. Using Bayes’ theorem the posterior can be written as
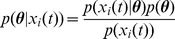
(3)where 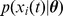 and 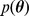 are the likelihood function and the prior distribution. The first takes into account the evidence in the data whereas the second expresses the uncertainty about model parameters before observing the data. Our choice of the prior distribution over model parameters takes into account the lognormal-like distribution of gene expressions observed in microarray and RNA-seq assays.

In particular, we considered gene-specific gaussian noise over predicted log expression levels with variance that is proportional to the variability over time in the data (**Text S1**). In this framework, log-expression profiles are fitted with the same relative error across different genes and the maximization of the posterior is equivalent to the minimization of the sum of squares
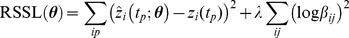
(4)where the sum over 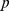 runs over the microarray time points, the sum over 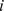 over the genes, and 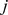 over the states; 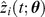 and 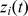 are the z-scores of 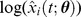 and 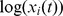 respectively. The first part of eq.(4) takes into account the goodness of the fit while the second part considers the penalty introduced by the prior. The parameter 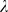 controls the extent of the penalization and was calibrated over the data.

Minimization of the RSSL (4) is a hard computational task given the high dimensionality of the data and model parameters as well as the non-linear analytical relationship between the population dynamics and rate parameters. We circumvented this problem by first determining the transition rates (which are common to the entire gene system) on a smaller subset of genes which captures the dynamical response of the whole system [3]. To select those representative genes, we clustered z-scores of log-transformed time-course data using k-means. Replacing each gene within a cluster with the gene that best represents that cluster, we obtained a less complex form for the RSSL, characterized by a lower number of parameters. We minimized such a reduced form to obtain transition rates and then we went on to minimize the original RSSL with those fixed transition rates on a per-gene basis. We considered an initial set of 256 representative genes after checking that starting from larger initial sets does not lead to different results in terms of population dynamics. Optimizations were performed in MATLAB R20012b with the function *lsqnonlin*. Further details concerning the identification of the initial set, the derivation of the reduced RSSL and the inference of parameters are given in the **Text S1**.

### Bayesian Model Selection

To address the selection of the number of states of the model, we trade off fit-to-data against model complexity by employing a quantitative Bayesian framework, which we illustrate here. First of all, the goal of this analysis is to obtain a posterior distribution over the models, i.e. over the number of states. Indicating with 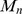 the model with 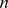 states, and considering log-transformed time-course data, 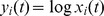, the posterior 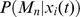 can be written as
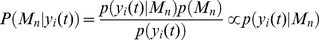
(5)where we have applied the Bayes theorem and we have considered a flat prior over models, and 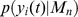 is the model marginal likelihood. In particular, we considered normal observational noise over 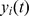 and chose weakly informative priors over model parameters (see **Text S1**). Introducing the extended set of parameters 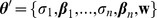, where 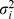 is the noise variance for gene 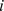, the marginal likelihood can be calculated as an integral over the parameters
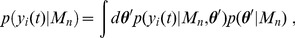
(6)where 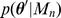 and 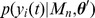 are the prior distribution of 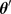 and the likelihood of the model respectively. Computing the marginal likelihood (6) is a hard computational task that involves the integration over the high dimensional space of the model parameters. As described in the following, we used a Markov chain Monte Carlo (MCMC) algorithm known as annealed importance sample (AIS) [15].

### Annealed Importance Sampling

The integral eq.(6) is very hard to compute given the high dimensionality of the integration domain. To perform the integration of our marginal likelihood we considered a MCMC algorithm known as annealed importance sampling (AIS) [15]. MCMC algorithms from statistical physics are widely used in Bayesian Inference [16], [17]. In particular, AIS combines the ideas of ‘annealing’ and ‘importance’ sampling providing low-variance unbiased estimators and enjoying an improved rate of convergence with respect to a naive MC scheme. In the following we very briefly outline the algorithm while for further details about the method we refer the interested reader to [15], [17], [18].

For simplicity of notation, in the following do not explicitly indicate the conditioning on the model 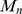. Let 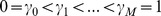 be a strictly increasing sequence of real numbers. The annealing bridges the prior and posterior distributions by introducing 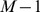 additional distributions: 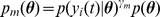. The inverse of 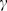 plays the role of a temperature. Let 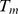 indicate the kernel of the Markov chain having 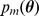 as equilibrium distribution. Using the AIS method, the marginal likelihood of eq.(6) can be written as
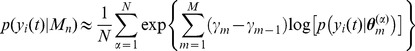
(7)where 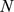 is the number of parallel chain runs, 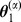 is sampled directly from the chosen priors whereas 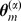 with 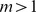 is sampled from 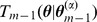. In order to update the model parameter values we considered a Metropolis-within-Gibbs scheme, which provides a faster rate of convergence with respect to a Metropolis-Hastings approach [19]. To test convergence, we looked at the evolution of our estimate of 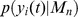 while increasing the number of parallel chains. Further details about the Gibbs sampling, our MCMC scheme and how we tested convergence are given in the **Text S1**.

### State Functional Signatures

To characterize the inferred states in terms of cell functional activities, we first identified the set of up-regulated genes for each state and then performed a functional enrichment analysis on those sets. A gene was defined as up-regulated in state 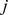 if its expression fold change between state 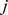 and state 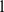 is greater than 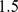. To perform the enrichment analysis, we used the standard DAVID (Database for Annotation, Visualization and Integrated Discoveries) software tool [20], [21] where we considered the whole set of noise-filtered genes (4960) as background and set the significance threshold at 5% FDR.

## Results

### Number of States

Our first aim was to quantify the most likely number of states that MCF-7 cells visit across 32 hours after estrogen stimulation. This is a model selection problem and requires finding the best trade-off of fit-to-data against model complexity. Indeed, while models with a few states give poor fits to the data, models with too many states risk to overfit by introducing artifactual states not bearing any biological information. To address the selection of the number of states, we first empirically examine how fit quality and overfitting depend on the number of states and second we trade off fit-to-data against model complexity by employing a quantitative Bayesian framework.

**Figure 2:**
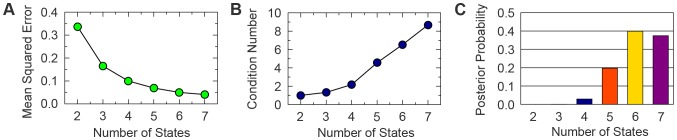
The number of single-cell states in the MCF-7 response to estrogen. (**A**) The mean squared error of the model fit to the microarray data decreases as function of the number of states: as expected, when the number of parameters increases, the quality of the fit improves. (**B**) The condition number is a measure of the similarity of the transcriptional profiles of the states. It increases as function of the number of states, 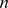, highlighting that over-fitting also increases with 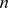. A good balance between fit quality and over-fitting must be found. (**C**) The model posterior probability, derived by a Bayesian approach, has a peak at 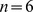, which shows that a model with six states strikes a good balance between fit-to-data and model parsimony.

In the empirical analysis, we looked at the mean squared error, i.e. the distance between the expression data and the model fitted values, and the degree of similarity between the different states for an 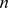-state model as a function of 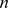 in order to monitor the fit-to-data performance and the overfitting respectively (**Fig. 2A,B**). More precisely, to empirically quantify the effects of overfitting, we recorded the condition number of the expression matrix 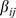, which is a measure of the similarity of the state profiles with each other, as a function of 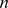 (**Text S1**). Above six states we observe marginal improvements in the quality of the fit and a steep increase in the similarity of the inferred states.

To confirm this empirical observation we considered the full Bayesian approach presented in Methods, which has been shown to be effective and principled approach to address model selection [17], [22], [23]. With this analysis we determine the posterior distribution of models over their number of states (**Fig. 2C**), i.e. the probability of having a certain number of states given the evidence in the data. While this is comparatively high for the five or the seven state model, interestingly, the posterior probability has a peak at six states. This suggests that a six-state model strikes a good balance between fit-to-data and model parsimony for the system considered here and in the following we present our findings obtained by employing such a model.

### Cell Transition Rates and Population Dynamics

**Figure 3:**
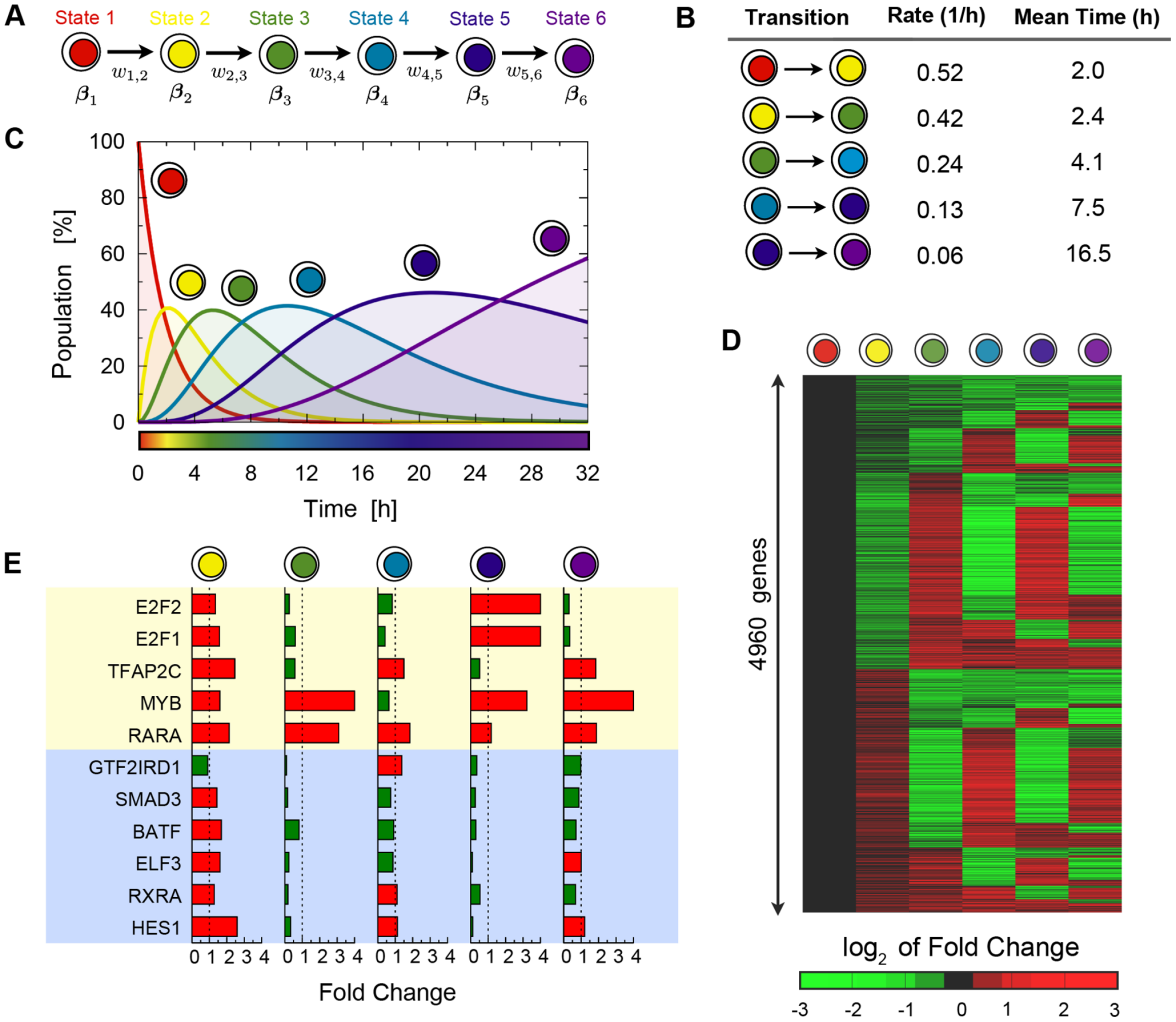
The single-cell transition rates and state transcriptional profiles in the MCF-7 response to estrogen. (**A**) Representation of the 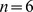 state model (chosen using our model selection methods, see **Fig. 2**) and its parameters, with the color code adopted in the following figures. (**B**) The predicted single-cell transition rates, 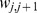 (and their inverse, the average times of transitions), between the different single-cell states. In the response to estrogen, the first state transition of cells appears to occur on a scale of about 2 hours, while the dynamics decelerates later on. (**C**) The predicted cell population dynamics, i.e., the fraction of cells in the different states as a function of time, 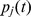. It emerges that after 32 hours (the duration of the microarray experiment) about 40% of the population has still not reached the final state. (**D**) Single-cell predicted transcriptional profiles (i.e., the 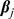 with 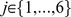). The heat map shows the log_2_ fold changes with respect to the first state. Large groups of genes are seen to change their expression from state to state. (**E**) Details of the state profiles (fold change) of the 11 primary transcription factor (PTF) genes identified in Ref. [4]. Genes highlighted in yellow (blue) are found in the microarray data to be on average up-regulated (down-regulated) after estrogen-stimulation during the time-course. As visible, a much finer expression pattern is revealed by the analysis of their expression across the single-cell states.

A pictorial representation of the six-state model is given in **Fig. 3A**, where the color code used in the rest of our paper figures is also set. **Fig. 3B–E** summarize its key parameters which we now discuss: the predicted state transition rates and the single-cell state genome-wide gene expression profiles.

We first discuss the model cell transition rates and the corresponding population dynamics, i.e., the single-cell events involved in the oestrogen response of the MCF-7 cells, and the resulting population behaviour. As can be seen by the average transition times (**Fig. 3B**), we find that the response of MCF-7 cells to estrogen is characterized by a faster early dynamics followed by a progressive deceleration. In fact, the transition from the initial state to the first intermediate state occurs with a mean time of 2.0 hours while the successive transitions are increasingly slower, the transition to the final state takes on average 16.5 hours. The population of state 1 is halved after roughly 2 hours of exposure to estrogen, while the fraction of cells being in state 1 becomes virtually 0 after 8 hours (**Fig. 3C**). States 2, 3, 4 and 5 peak respectively at 2, 5, 10 and 20 hours. We find that at 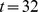 hours only 58% of cells have reached the sixth, final state while 36% and 6% of cells are still respectively in states 5 and 4.

**Figure 4:**
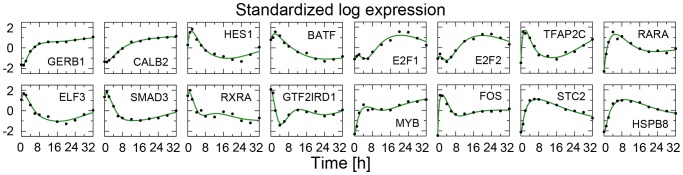
Fits to gene expression time-course data. The fit to some key genes, comprising the 11 primary transcription factors identified by Cicatiello et[4] and other important estrogen-responsive genes [1], [2], are shown: black circles represent time-course (standardized) data while green lines represents the gene expression predicted by the six-state model.

To provide an example of the quality of our fitting procedure, in **Fig. 4** we show the fit to 16 genes comprising the 11 primary transcription factors identified by Cicatiello et al. [4] and other important estrogen-responsive genes [1], [2].

#### The ZR-75.1 cell line system

**Figure 5:**
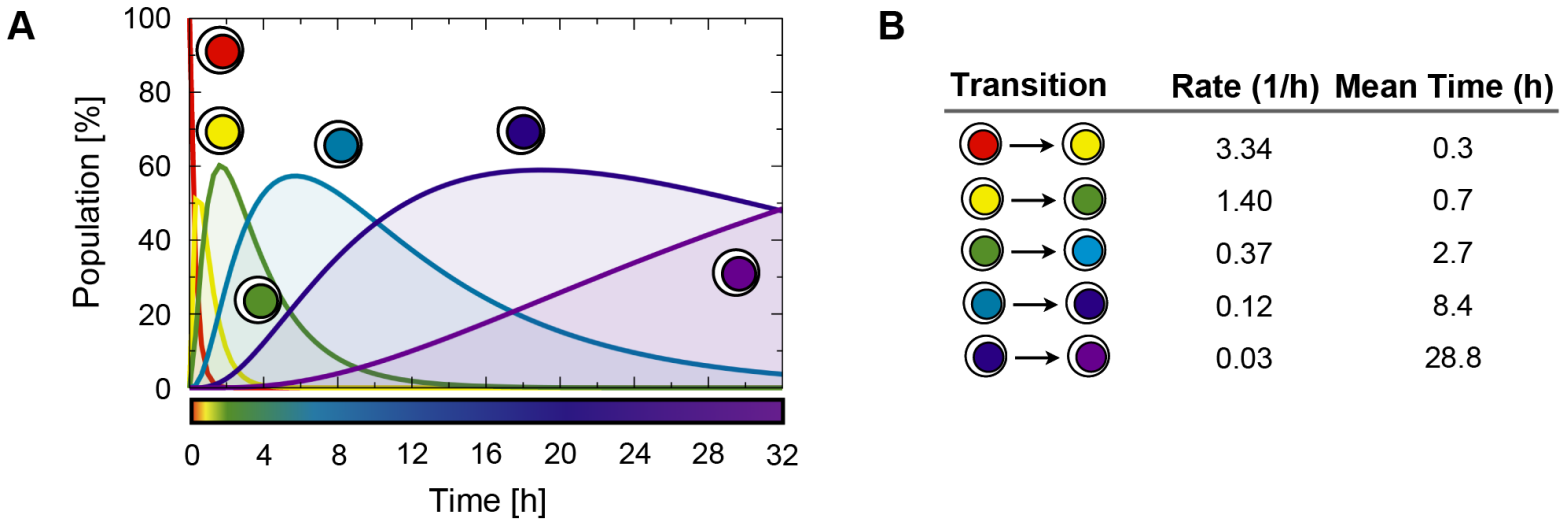
The single-cell transition rates in the ZR-75.1 system. Results of the six-state model for time course data in hormone-starved ZR-75.1 cells responding to estrogen stimulation are shown for comparison with the MCF-7 system of **Fig. 3**. (**A**) Cell population dynamics. (**B**) Rates and mean times of transitions. In ZR-75.1 the response to estrogen is initially one order of magnitude faster than in MCF-7.

The other model of breast cancer cells studied by Cicatiello et al. [4], ZR-75.1, is known to share with MCF-7 a similar, yet faster transcriptional response to estrogen, including, for example, cyclin genes and thus reflecting a more rapid cell cycle start and progression [4], [24]. We applied the six-state model to the ZR-75.1 dataset as well and found a very good fit, highlighting, indeed, a faster dynamics for earlier states in ZR-75.1 in comparison to MCF-7. For instance, the first transition takes places in only 0.3 hours rather than 2 hours (**Fig. 5**).

### Single-Cell State Transcriptional Profiles

State-specific gene expressions, i.e., the transcriptional profiles of the states, were determined for all the 4960 noise-filtered genes differentially expressed during the time-course assay of Ref. [4], by use of the fitting procedure described in the Methods section. In **Fig. 3D** the fold change of the whole set of genes is shown across the six states: in a given state, genes in red (green) are up-regulated (down-regulated) with respect to state 1. **Fig. 3D** also highlights that the states have very distinct transcriptional signatures, with a substantial fraction of genes (around 50%) changing from up- to down-regulated with respect to the neighboring states.

**Fig. 3E** collects a subgroup of genes of **Fig. 3D**: it shows the details of the state profiles of the 11 primary transcription factor (PTF) genes highlighted in Ref. [4]. In that work, it was found that 5 PTF genes (marked by a yellow background in **Fig. 3E**) were on average up-regulated after estrogen stimulation during the time-course, whereas the other 6 (marked with a blue background) were down-regulated. Our analysis is consistent with such an overall observation, however, it reveals finer details of the expression behavior of those genes across the different states, which are more complex than either a simple up-regulation or down-regulation. For instance, the two transcription factors E2F2 and E2F1 are found to peak at state 2 and 5, but are otherwise down-regulated.

#### Marker Genes

**Figure 6:**
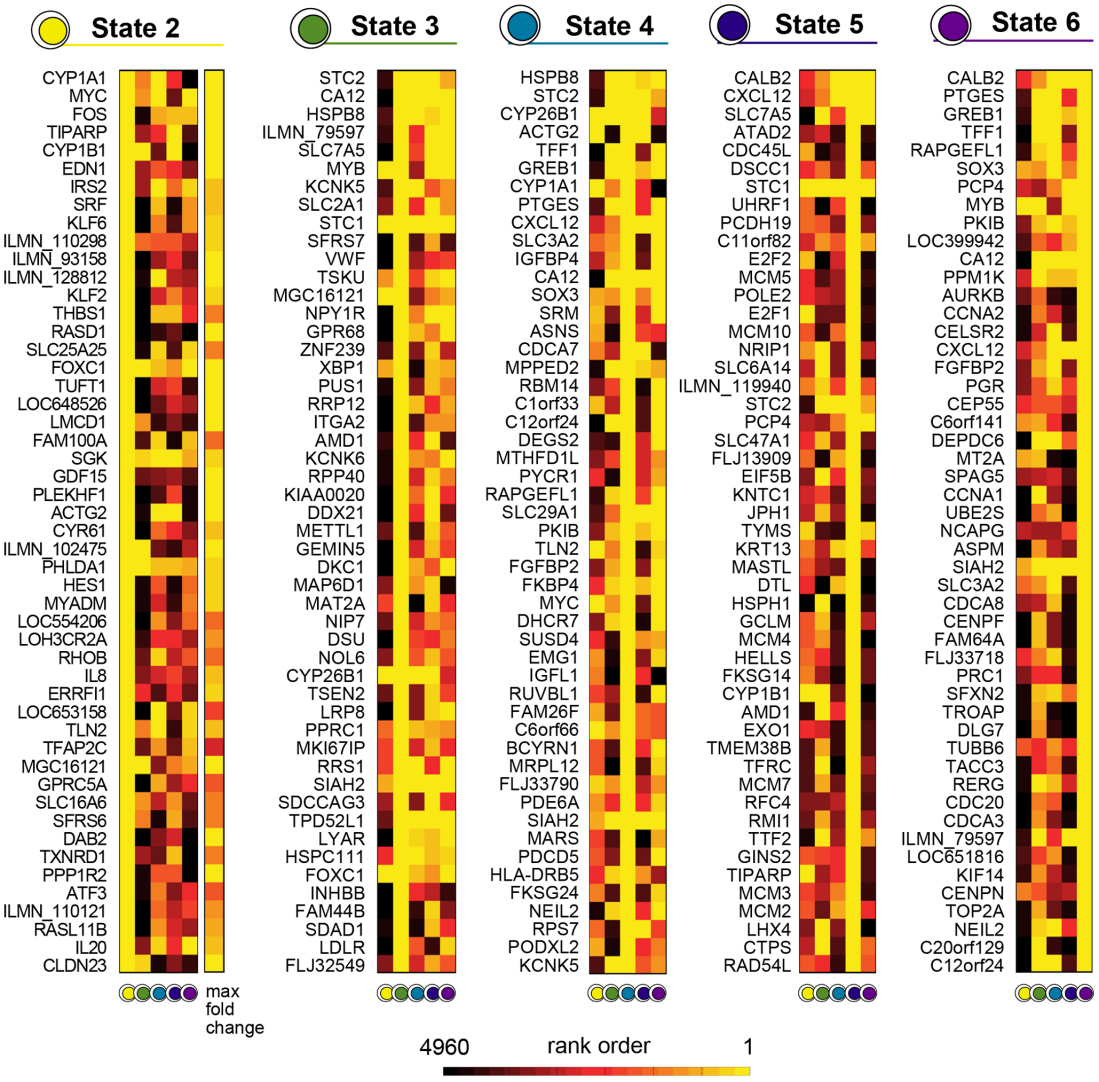
Marker genes in the MCF-7 system. In each state of a six-state model, genes are ranked by their state-expression fold change with respect to the first state. Here, only the top 50 are shown along with their ranking in the other states. For the top genes of state 2 also the rank assigned considering a maximum fold change criterion over the time course is shown for comparison (separated column). The state-based ranking criterion highlights marker genes which would otherwise pass unnoticed.

We also identified the state-specific marker genes. In each state, genes were ranked by their fold change with respect to state 1. In **Fig. 6** we list the top 50 ranked genes in each state and we show for comparison also their rank in the other states. Ranking based on the state features is different from other, more conventional criteria. For example, for the top genes of state 2 we also show their ranks assigned with respect to their maximum fold change across temporal expression profiles, defined as the ratio of the maximum to the minimum expressions across the time profile. There are some genes having a low-medium rank with respect to the maximum fold change, but with a very high rank using our criterion. The behaviour of a few important marker genes are illustrated in the Discussion section below.

#### Primary genes

**Figure 7:**
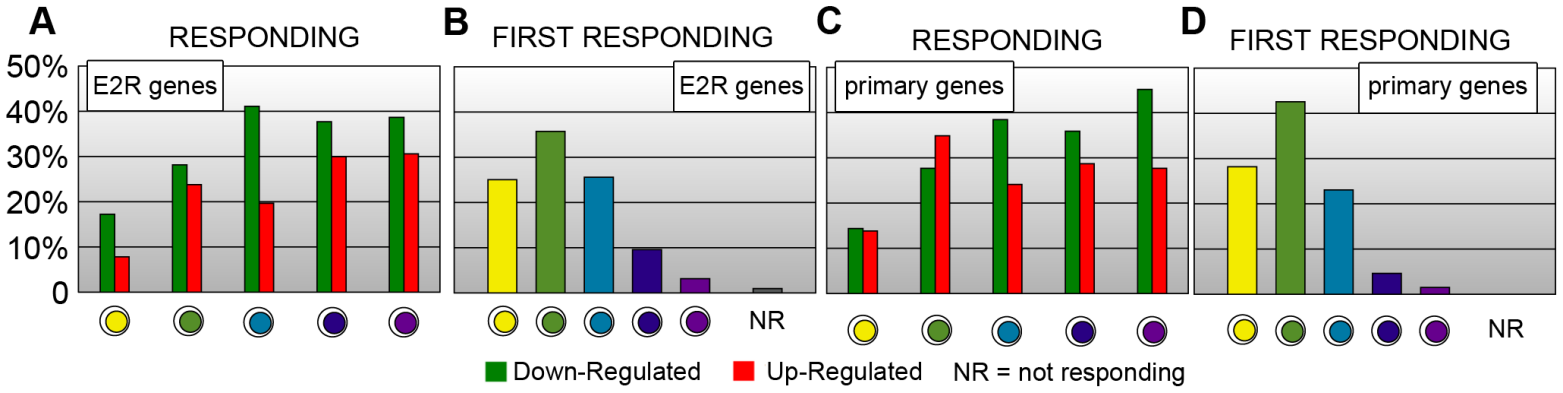
Estrogen responding genes per state. Among the entire gene set considered in the MCF-7 cell experiment, 1270 also responded in ZR-75.1 cells. These are referred to as common ‘estrogen-regulated genes’ (E2R genes) in [4]. ‘Primary genes’ are their subgroup having a ER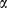 transcription factor binding site within 10 kb around the TSS. The figures show how E2R and primary genes are responding across the single-cell states of a six-state model. (**A**) Fraction of up-regulated and down-regulated E2R genes. (**B**) Fraction of first-responding E2R genes, i.e., of genes that respond for the first time in a given state. (**C**) and (**D**) show the analogous pattern of primary genes.

We also looked at the response of important set of genes identified in the original paper by Cicatiello et al. [4]. In particular we considered the set of 1270 genes responding to estrogen in both MCF-7 and ZR-75.1 cell lines (named common ‘estrogen regulated genes’ (E2R)), and its subset of 218 primary genes (i.e., the subset having a ER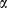 transcription factor binding site within 10 kb of the TSS). We show the fraction of responding up-regulated and down-regulated genes across the single-cell states in those two sets (**Fig. 7A,C**). The two groups of genes have very similar trends: the number of genes either up-regulated or down-regulated increases in successive states, as expected, and the fractions of down-regulated genes are almost always larger than those of up-regulated ones. In **Fig. 7B,D**, we show, for the two sets, the fraction of genes that first-respond in each state. In our notation, a gene is ‘responding’ if it is either up-regulated or down-regulated whereas it is ‘first-responding’ in state 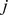 if it responds in state 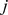 but it has not responded in any previous states. More than 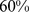 of genes have responded first in states 1 and 2 in both sets. The characteristics of a few specific primary genes are illustrated in the Discussion section below.

### State Functional Signatures

**Figure 8:**
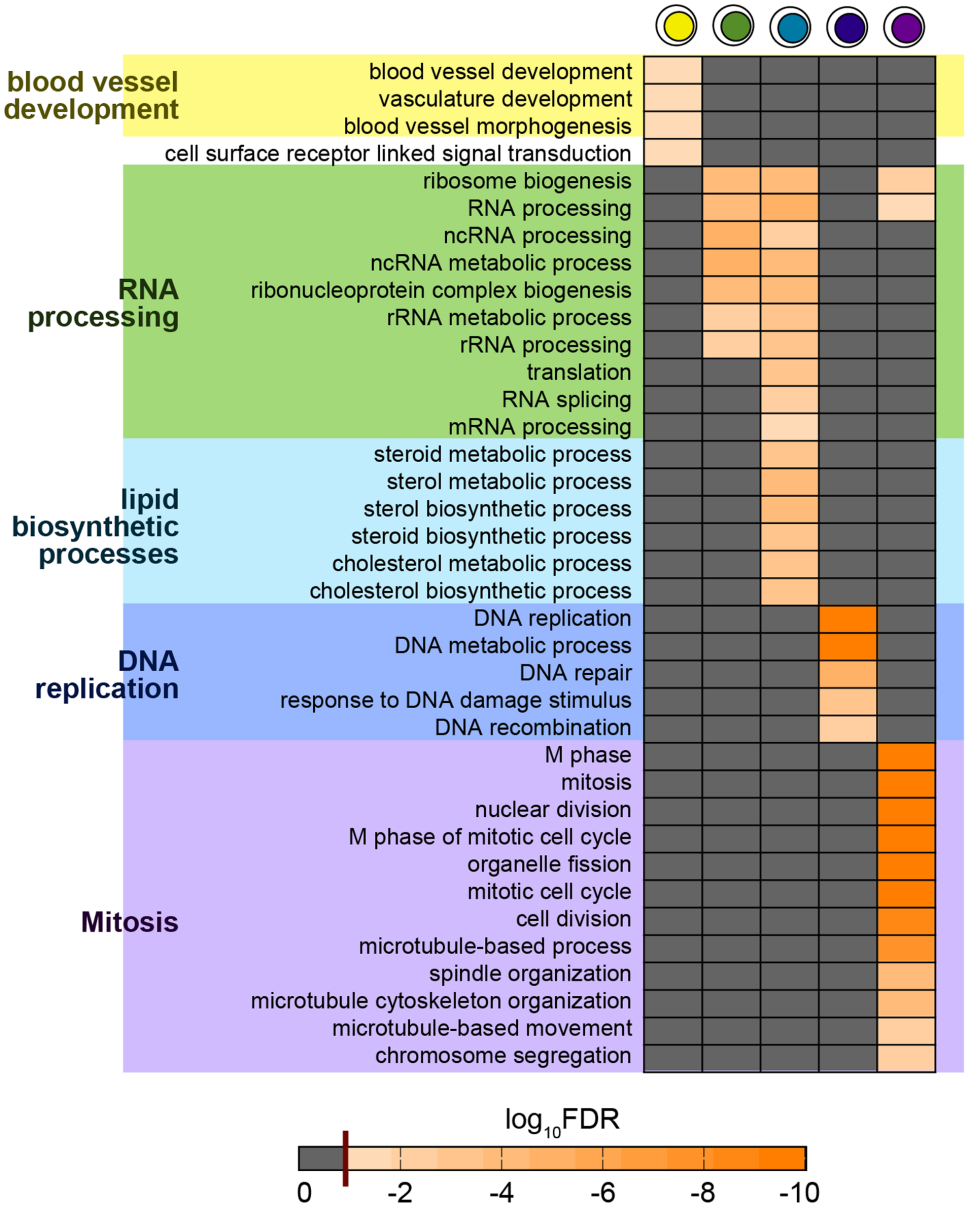
Single-cell state functional signatures in the MCF-7 system. The GO terms enriched among up-regulated genes in each single-cell state are shown (in the six-state model). Only terms that are significant within 5% false discovery rate (FDR) are shown (the 5% FDR threshold is shown as dark red line in the bar). Terms in grey are not significantly enriched. The processes activated in the sequence of states appear to well describe the events characterizing the mitogenic response associated to ER-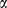.

In order to characterize the biological functional signatures of the predicted single-cell states, we conducted a state-specific enrichment analysis of GO terms [25] proceeding as discussed in Methods. The biological processes significantly enriched in the different states (**Fig. 8**) are found to be strikingly well linked to the mitogenic effects of estrogens, whose cascade can be here dissected across the specific states. This is illustrated in the Discussion section below. These results confirm that the inferred cellular states capture timing and nature of known cellular responses to estrogen and provide a more detailed view of the dynamics of these processes.

## Discussion

Current high-throughput RNA profiling techniques, such as microarray and RNA-seq, provide the tools to study cell transitions on a genome-wide scale. However, they return data averaged over heterogeneous populations, hiding the possibility to characterize expression at the single-cell level. High-throughput single-cell assays are being currently developed but are still in their initial stages. Thus, we considered here a general quantitative model [3] that allows reconstructing from population-averaged time-course data, e.g., microarray data, a genome-wide characterization of the dynamics of single cells. The model describes, via Markov processes, the scenario where cells undergo stochastic transitions across multiple states. By fitting time-course data, the expression signatures of the states that cells visit during their transitions and the rates that characterize such transitions are then derived.

Here, we have employed such an analysis method to investigate, in particular, the estrogen response of hormone-starved MCF-7 cells, a model of breast cancer widely used to characterize the estrogen response in breast tumors [26]–[28]. We considered one of the largest available microarray dataset [4] on hormone-responsive genes identified in human breast cancer cells. Our findings are fully consistent with previous results, and we reveal new insights on the transcriptional dynamics at the single-cell level in the response to estrogen. Although time-course epigenetic data and other time-course data, when available, can be included in the model [3] and important pieces of information, such as chromatin three-dimensional folding and organization [9], could be taken into consideration, we here have only considered microarray time-course data. Furthermore, in the nucleus and cytoplasm of real cells, a number of other effects and complications arise which are likely to play important roles on the system behavior as found in the study of other complex fluids (see, e.g., [29]–[33] and ref.s therein).

Nevertheless, in the simplified framework considered here, we have shown that the dynamic estrogen response of MCF-7 cells can be described using six single-cell states across the 32 hours after stimulation. The dynamics across those states is characterized by a faster early response to the initial stimulus, occurring on a scale of 2 hours, followed by a progressive deceleration of the transitions (**Fig. 3B,C**): at 32 hours 40% of the population is still not in the mitotic state, which is the last state in this description.

Our analysis has derived the genome-wide transcriptional profiles of the states (**Fig. 3D**), revealing the fine details of the expression behavior across the different states. A focal case study has been the group of the 11 primary transcription factor (PTF) genes highlighted in Ref. [4]. For instance, we find that E2F2 and E2F1 peak at state 2 and 5, but are otherwise down-regulated (**Fig. 3E**). In estrogen-responsive BC cell lines, these proteins are able to promote G1-S transition [24], [34] and their overexpression causes hormone-independent proliferation and antiestrogen-resistance [35]. An other example is up-regulation of the retinoic acid receptor subtype RARA in states 2–5 with respect to state 1 (**Fig. 3E**). This confirms its high expression in ER-positive BC cells, where the protein encoded by this gene has been shown to accumulate as consequence of ER-mediated trans-activation of the RARA-1 gene promoter [36]. The overlapping between RARA binding sites and those of ER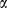 throughout the genome results in crosstalk between this two molecules leading to the regulation of cancer-associated genes [37].

We also identified the genes marking the inferred states (**Fig. 6**). In our top 50 ranked state-specific marker genes, we find many genes known to play a key role in the estrogen response, the hormone-responsive breast cancer phenotype and tumor response to endocrine therapy. Among all, it is worth mentioning the FOS and MYC genes, top rank members in state 2, that are known to promote cell replication in response to extracellular signals, including estrogen, driving quiescent cells into the cell cycle, activating key cell cycle genes such as cyclins D1, D2, E and A, CDK4, E2F1 and E2F2. The same is true for TFF1 and GREB1 in state 4. The TFF1/ps2 protein is a member of the trefoil protein family, found to be expressed in human breast carcinomas and involved in controlling expansion or contraction of the ductular system through its mitogenic properties. The ATF/CREB family plays a role in breast cancer and is considered to be an effective therapeutic target gene. Some members of this gene family are protective against breast cancer but others such as ATF4, ATF5, and CREB, promote breast cancer pathology. In fact, CREB can contribute to malignancy of breast epithelia inducing transcription of aromatases that, in turn, lead to increased estrogen levels establishing a vicious cycle in the tissue. As an example of positive feedback regulation, estrogen causes CREB to bind and activate the cyclin D1 promoter [38]. By activating cyclin D1, which causes cells to progress through the cell cycle, activation of CREB represents a central event in phase transitions. Furthermore, dominant negative CREB has been shown to block the transcription of the estrogen-responsive BCL-2 gene in MCF7 cells [39]. Since this protein blocks apoptosis, this suggests an additional role for a mid-G1 event. The few examples considered here suggest the utility of our model in identifying genes playing a critical role in breast cancer development and progression and the time of their action. Moreover, we find that the model identifies state-specific genes that would have been ignored by considering a standard criterion as maximum fold change. This is the case of TFAP2C, whose overexpression highlights the key role in invasive breast cancer correlating with a poorer response to anti-hormone therapy and reduced patient survival [40].

Our functional enrichment analysis of state-specific GO terms provides a full characterization of the inferred states (**Fig. 8**). Our findings are not only consistent with the known picture of estrogen acting as potent mitogen, but they provide, for the first time, new insights on the cellular functional activities at the single-cell level. In particular, the terms enriched in state 2 are involved in angiogenesis, which could be associated with the in vivo phenomenon of the “angiogenesis switch” [41], an alteration in the balance of naturally occurring endothelial growth factors and inhibitors [42]. Following the switch to an angiogenic phenotype, endothelial cells must then proteolytically degrade the extracellular matrix that surrounds them, migrate and proliferate, form capillary structures, and anastamose into a vascular network that characterizes the transition of a tumor from a dormant state to a malignant state [43]. A variety of activities related with RNA processing becomes enriched in state 3 and persists in state 4, including processing of non-coding RNAs. State 4 is also characterized by lipid metabolic and biosynthetic processes. DNA replication, repair and recombination mark state 5, which coincides with the S phase, while in state 6 fully fledged mitosis related terms are enriched. The emerging picture is that state 2, 3 and 4 correspond to three subphases of the G1 phase of the cell cycle, whereas states 5 and 6 can be more directly associated respectively to the S phase and mitosis. The functional roles assigned to the different predicted states are fully consistent with previous studies on the estrogen response of MCF-7 cells, and they match well the known intervening phases of the cell cycle. For example, we find that more than 40% of the entire population is in interphase between 16 and 28 h, which is precisely the time that MCF-7 cells need to enter the S phase [4], [24], [44]. Moreover, in previous studies [4] it was noticed that genes involved in mitosis show maximal response between 28 and 32 h.

Interestingly, the single-cell states identified here are consistent with the ratchet-like model proposed for estrogen stimulation of cell proliferation, which foresees a permissive effect of the hormone on multiple, sequential cell cycle restriction points [45]. Indeed, our method allowed identification of estrogen target genes involved in each of these transitions, providing for the first time a genetic explanation also for the dynamics of breast cancer cell responses to antiestrogen drugs, such as those described for the selective estrogen receptor down-regulator ICI 182,780/Faslodex, a pure antihormone used for treatment of these tumors [28]. In this respect, these results identify multiple genes whose mutation might cause perturbation of one or more cell states resulting in estrogen-independent cell cycle progression, one of the key events believed to cause the resistance to antihormones observed in 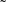
30% breast cancer patients undergoing these therapies.

In conclusion, we have considered a stochastic model of the events characterizing single-cell transitions during the estrogen response of a breast cancer model, MCF-7 cells. Our methods allow to identify the single-cell states intervening during the processes, by using only population-averaged time-course data, such as microarray or bulk RNA-Seq data. Interestingly, it recapitulates the key known biology facts about the system and, for the first time, sheds light on the single-cell events and on the states transversed in the process. Our approach could be similarly applied to other cell transitions and could easily accommodate additional data types, such as epigenetic data.

## Supporting Information

Text S1 **Supplementary methods section presenting in greater detail the model, parameter estimation and the Bayesian framework employed for model selection.**
(PDF)
