# Supplementary material for: Single-Cell States in the Estrogen Response of Breast Cancer Cell Lines
Source: PLoS One. 2014 Feb 25;9(2):e88485. doi: 10.1371/journal.pone.0088485 (PMC3934861; doi:10.1371/journal.pone.0088485)
Supplement: Text S1 — Supplementary methods section presenting in greater detail the model, parameter estimation and the Bayesian framework employed for model selection. (PDF) [file pone.0088485.s001.pdf]

Supplementary Text for "The Single-Cell States in the Estrogen Response of Breast Cancer Cells"

Francesco Paolo Casale, Giorgio Giurato, Giovanni Nassa, Jonathan W. Armond, Chris J. Oates, Davide Corá, Andrea Gamba, Sach Mukherjee, Alessandro Weisz, Mario Nicodemi

Contents

1 Introduction **1**

2 Model and Model Fitting **2**
2.1 The Model 2
2.2 Solution of the Master Equation 2
2.3 Maximum-a-Posteriori framework 3
2.4 Model fitting 4

3 Model Selection **6**
3.1 Fit quality and overfitting 6
3.2 Bayesian Framework 7
3.3 Annealed Importance Sampling 8

1 Introduction

In this additional part of our manuscript we provide supplementary information about the materials and methods concerning the inference of model parameters and the Bayesian model selection approach we used to determine the most likely number of states. As discussed in the main text, the biological system consists of hormone-starved MCF-7 and ZR-75.1 exposed to estrogen across 32 hours. Here we consider microarray time-course data from Cicatiello et al. [1] which consist of expression profiles taken at 12 time points, $t = 0, 1, 2, 4, 6, 8, 12, 16, 20, 24, 28, 32$, after estrogen-stimulation. Data were preprocessed as described in Cicatiello et Al. [1] and summarized in the main text (Methods). Considering only genes with a detection p-value≤ 0.05 and ILLUMINA DiffScore ≤ -40 or

≥ 40, 4960 and 4106 estrogen responsive genes were identified respectively in MCF-7 and ZR-75.1.

2 Model and Model Fitting

In this section, we describe the model used in the main text [2], and how we fitted it to the data. In particular, we discuss how we dealt with the high dimensionality of the data and the regularisation that we considered in our maximum-a-posteriori (MAP) approach.

2.1 The Model

Our time-course data comprise $k = 4960$ noise-filtered genes over $T = 12$ time points, $t = 0, 1, 2, 4, 6, 8, 12, 16, 20, 24, 28, 32$. The dataset consists, thus, in a $k \times T$ matrix, where $x_i(t)$ represents the measured expression of gene i at time t.

As described in the main text, a model with n states has $n - 1 + nk$ parameters: $n - 1$ transition rates, and kn state-specific expression parameters defining the states. Let us define the rate vector $\mathbf{w} = (w_{1,2}, ..., w_{n-1,n})$, where $w_{j,j+1}$ is the transition rate from state j to $j + 1$, and indicate with β_{ij} the expression of gene i in state j. The population dynamics is completely defined by transition rates through the master equation of our Markov model

$$\frac{\mathrm{d}p_j(t)}{\mathrm{d}t} = w_{j-1,j}p_{j-1}(t) - w_{j,j+1}p_j(t) \qquad j = 1, ..., n-1 \tag{1}$$

where $p_j(t)$ indicates the fraction of cells in state j at time t and $w_{0,1}$ and $w_{n,n+1}$ are defined to be zero. The master equation can be exactly integrated and the analytic form of $p_j(t)$ can be obtained (see next subsection). We assume that the cell population is homogeneous at $t = 0$.

Gathering the parameters within the multidimensional variable $\boldsymbol{\theta} = \{\mathbf{w}, \boldsymbol{\beta}_1, ..., \boldsymbol{\beta}_n\}$, the predicted population-averaged expression $\hat{x}_i(t; \boldsymbol{\theta})$ of gene i at time t is given by

$$\hat{x}_i\left(t; \boldsymbol{\theta}\right) = \sum_{j=1}^{n} p_j\left(\mathbf{w}; t\right) \beta_{ij} \tag{2}$$

where we have explicitly shown the dependence of the probabilities on the transition rates.

2.2 Solution of the Master Equation

The master equation eq.(1) is a system of $n - 1$ independent (n minus one normalization condition) linear ordinary differential equations and we want to solve it with initial conditions $(p_1(0), p_2(0)..., p_{n-1}(0)) = (1, 0, ..., 0)$. This Cauchy problem can be fully solved:

it is straightforward to determine $p_1(t)$ from the first equation, while by induction it can be shown that

$$p_j(t) = \left(\prod_{i=1}^{j-1} w_i\right) \sum_{i=1}^{j-1} \frac{\exp(-w_i t) - \exp(-w_j t)}{\prod_{k\neq i}^{n} (w_j - w_i)} \quad (3)$$

Finally, $p_n(t)$ can be determined by imposing the normalization $\sum_j p_j(t) = 1$.

For example, for a 4 state model we have

$$p_1(t) = e^{-w_1 t} \quad (4)$$

$$p_2(t) = \frac{w_1}{w_2 - w_1}\left(e^{-w_1 t} - e^{-w_2}t\right) \quad (5)$$

$$p_3(t) = \frac{w_1 w_2}{w_2 - w_1}\left(\frac{e^{-w_1 t} - e^{-w_3 t}}{w_3 - w_1} - \frac{e^{-w_2 t} - e^{-w_3 t}}{w_3 - w_2}\right) \quad (6)$$

$$p_4(t) = 1 - p_1(t) - p_2(t) - p_3(t) \quad (7)$$

The analytical solution (3) may become computationally unstable for $w_i \approx w_j$ with $i \neq j$. So we also exploit the formal solution of equation (1) which can be written in the matrix form:

$$\frac{dp(t)}{dt} = Ap(t) \quad (8)$$

where

$$p(t) = \begin{pmatrix} p_1(t) \\ p_2(t) \\ ... \\ p_{n-1}(t) \end{pmatrix}, \quad A = \begin{pmatrix} -w_1 & 0 & ... & 0 \\ w_1 & -w_2 & ... & 0 \\ ... & & & \\ 0 & 0 & ... & -w_{n-1} \end{pmatrix} \quad (9)$$

Although the solution $p(t) = e^{At}p(0)$ is numerically stable, matrix exponential is computationally challenging. We therefore considered the matrix form only in regions of the domain where the analytical solution might be unstable.

2.3 Maximum-a-Posteriori framework

As described in the main test, we inferred the model parameters using a maximum-a-posteriori (MAP) approach.

Let $y_i(t)$ denote the log-transformed time-course data and $\hat{y}_i(t)$ denote model predictions for $y_i(t)$. We considered gene-specific normal distributed model errors $y_i(t) = \hat{y}_i(t;\boldsymbol{\theta}) + \epsilon_i$ where $\epsilon_i \sim \mathcal{N}(0,\ \sigma_i^2)$. Moreover, we suppose that the gene specific noise is proportional to the gene standard deviation across measured time points, $\sigma_i = \eta\,\mathrm{std}\left(\{y_i(t_p)\}_p\right)$, where $\{t_p\}_p$ denote the microarray time points. By introducing the z-scores $z_i(t;\boldsymbol{\theta}) = \frac{y_i(t;\boldsymbol{\theta}) - \mathrm{mean}\left(\{y_i(t_p)\}_p\right)}{\mathrm{std}\left(\{y_i(t_p)\}_p\right)}$ and $\hat{z}_i(t;\boldsymbol{\theta}) = \frac{\hat{y}_i(t;\boldsymbol{\theta}) - \mathrm{mean}\left(\{y_i(t_p)\}_p\right)}{\mathrm{std}\left(\{y_i(t_p)\}_p\right)}$ the likelihood can be written as

$$L\left(\boldsymbol{\theta}|z_i(t)\right) \propto \exp\left(-\frac{\sum_{ip}(\hat{z}_i(t_p;\boldsymbol{\theta}) - z_i(t_p))^2}{2\eta^2}\right) = e^{-\frac{kT}{2\eta^2}\cdot\mathrm{RSS}(\boldsymbol{\theta})}. \quad (10)$$

where we have introduced the residual sum of squares

$$\mathrm{RSS}\left(\boldsymbol{\theta}\right)=\frac{1}{kT}\sum_{ip}\left(\hat{z}_i\left(t_p;\boldsymbol{\theta}\right)-z_i(t_p)\right)^2 \tag{11}$$

In a MAP approach, the goal is the maximization of the posterior distribution $p(\boldsymbol{\theta}|z_i(t))=\frac{L(\boldsymbol{\theta}|z_i(t))p(\boldsymbol{\theta})}{p(z_i(t))}$ where $p(\boldsymbol{\theta})$ is the prior distribution over the model parameters and $p(z_i(t))$ is a normalization constant. We considered flat priors over rate transitions while for expression parameters we considered

$$p(\boldsymbol{\theta})\propto\exp\left\{-\frac{\sum_{ij}(\log(\beta_{ij}))^2}{2\Sigma^2}\right\}, \tag{12}$$

which resembles the lognormal distributions observed in microarray and RNA-seq assays for gene expressions. The posterior distribution can be thus written as

$$p(\boldsymbol{\theta}|z_i(t))\propto L\left(\boldsymbol{\theta}|z_i(t)\right)p(\boldsymbol{\theta})\propto\exp\left\{-\frac{kT}{2\eta^2}\cdot\mathrm{RSSL}(\boldsymbol{\theta})\right\} \tag{13}$$

where we have introduced

$$\mathrm{RSSL}\left(\boldsymbol{\theta}\right)=\mathrm{RSS}\left(\boldsymbol{\theta}\right)+\frac{\lambda}{kT}\sum_{ij}\left(\log\beta_{ij}\right)^2 \tag{14}$$

The parameter $\lambda=\left(\frac{\eta}{\Sigma}\right)^2$ controls the extent of the penalization for either very high or very low expression levels. To maximize the posterior (13), we minimized the non-linear sum of squares $\mathrm{RSSL}(\boldsymbol{\theta})$.

2.4 Model fitting

The very high dimensionality of the dataset ($kT=10^4\div10^5$) and the strong non-linear dependence of probabilities $p_j(\mathbf{w};t)$ on rate parameters $\mathbf{w}$ (see equation (3)) makes the minimization of RSSL a hard computational task. From (2) it can be clearly seen that transition rates $\mathbf{w}$ are parameters common to all the expression levels predicted by the model. We thus broke the minimization into two parts: we first reduced our analysis to a subset of k_1 representative genes to determine $\mathbf{w}$; once $\mathbf{w}$ is determined, the minimization can proceed gene-by-gene with fixed rates to fit the whole dataset.

In order to determine transition rates we performed k-means clustering on standardized data to obtain k_1 clusters of genes, and then we confused each gene with the gene closest to the centroid of its cluster. Doing so, we obtained the following reduced form for the RSSL

$$\mathrm{RSSL}\left(\boldsymbol{\theta}\right)\approx\mathrm{RSSL}_{k_1}^{(\mathrm{R})}\left(\boldsymbol{\theta}^{(\mathrm{R})}\right)=\frac{1}{T}\sum_{\alpha p}f_\alpha\left(\hat{z}_{m_\alpha}\left(t_p;\boldsymbol{\theta}^{(\mathrm{R})}\right)-z_{m_\alpha}(t_p)\right)^2+\frac{\lambda}{T}\sum_{\alpha j}f_\alpha\left(\log\beta_{m_\alpha j}\right)^2 \tag{15}$$

where m_α denotes the index of the gene closest to the centroid of cluster α, f_α denotes the fraction of genes contained in cluster α and $\boldsymbol{\theta}^{(\mathrm{R})} = \{\mathbf{w}; (\beta_{m_\alpha j}) \in \mathbb{R}_{k_1,n}\}$ denotes the reduced set of model parameters. Clustering was performed on Matlab using 100 replicates after filtering out 25% of genes with lowest standard deviation across time. This further cut was performed to avoid amplifying uninformative genes with very low standard deviation while considering z-scores and affecting transition rate estimates. Those genes where then reintroduced in the analysis in the gene-by-gene minimizations.

To select the appropriate number of representative genes we minimized the system for different values of k_1 and monitored changes in the population dynamics. Population dynamics did not significantly change considering more than 256 clusters, as shown in **Figure S1** where the number of representative genes is doubled from 256 to 512.

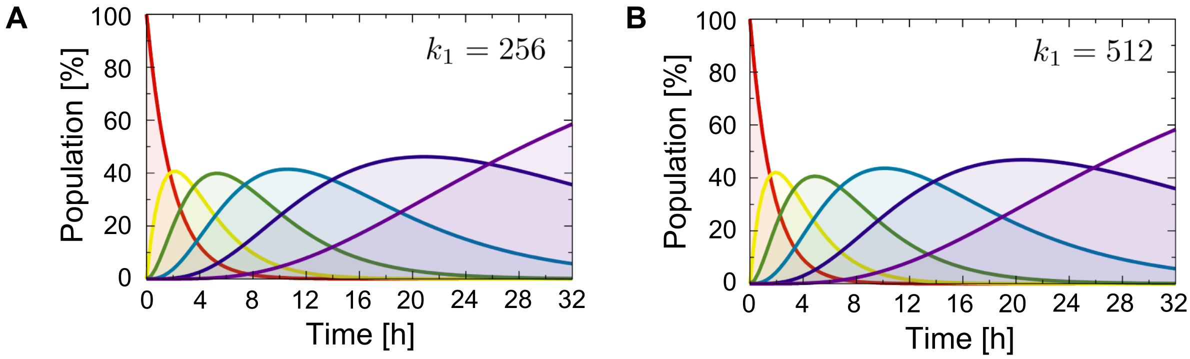

Figure S1. Population dynamics obtained by considering different numbers of initial genes. Differences in the population dynamics inferred for $k_1 = 256$ **(A)** and $k_1 = 512$ **(B)** are hardly noticeable.

Non-linear Least Squares Fitting was done in MATLAB R20012b using the function *lsqnonlin* (widely used to solve non-linear least square minimizations), which is based on a trust region algorithm.

We considered $\lambda = 0.01$ that corresponds to a weakly informative prior over expression levels. This regularization improves reproducibility and rate of convergence. We also checked that our choice of priors does not affect too much the estimated values of model parameters. To do so, we compared our results with those obtained though a maximum-likelihood (ML) approach ($\lambda = 0$). The minimisation for $\lambda = 0$ was performed using as starting point the minimum obtained for $\lambda = 0.01$. As shown in **Figure S2**, we find very good concordance both in the population dynamics and expression levels.

In order to assess the convergence of the algorithm and the convexity of the posterior, we considered 100 different starting points and checked convergence to the same value by following the evolution of each separate run. In **Figure S3** we show the mean of RSS, RSSL and w_1 as function of the number of function evaluations of the trust region algorithm for the case $n_s = 6$. Random initial points were selected as follows: initial rates

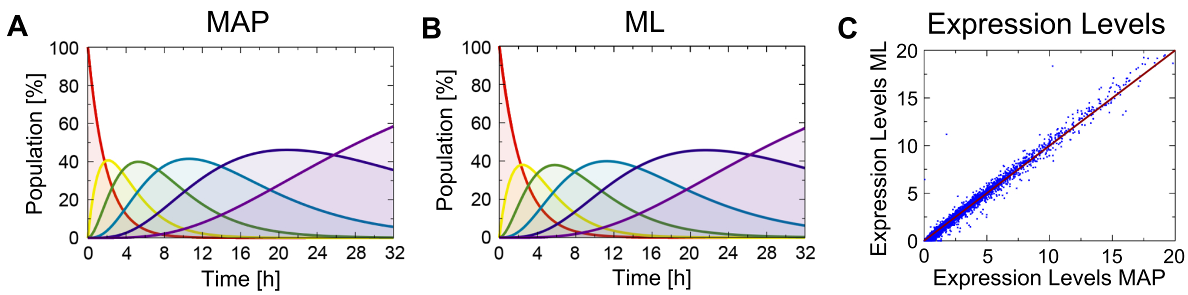

Figure S2. Comparison between the minimum obtained through MAP ($\lambda = 0.1$) and ML ($\lambda = 0$). Population levels obtained through MAP **(A)** and ML **(B)** are almost identical. Apart from very rare exceptions, a very good accordance is found also for expression levels **(C)**.

were taken uniformly between 0 and 1 [1] whereas state signatures parameter were sampled from a large lognormal distribution. The results of the fits are given in the Main Text.

3 Model Selection

In this section we discuss the Bayesian approach to determine the most likely number of states describing the estrogen-response of MCF-7.

3.1 Fit quality and overfitting

Following the procedure we presented in the previous paragraph, we fitted models with up to 7 states. We found that models with few states fit poorly the data while models with more states can fit very well the temporal expression profiles (see **Fig.4** of the Main Text). As we can see the fit improves while considering more state, but the extent of the improving is less and less significative. Models with too many states, however, may overfit the data, given their large number of parameters. We used the condition number of the expression matrix relative to the selected k_1 representative genes as a rough estimate of the overfitting. Equation (2) can be written in the matrix form $BP(t) = \hat{X}(t)$ where we have introduced the matrices

$$P(t) = \begin{pmatrix} p_1(t) \\ \dots \\ p_n(t) \end{pmatrix} \quad , \quad \hat{X}(t) = \begin{pmatrix} \hat{x}_1(t) \\ \dots \\ \hat{x}_n(t) \end{pmatrix} \quad \text{and} \quad B = (\beta_{ij}) \, , \tag{16}$$

The condition number of B is defined as the ratio of the maximal and minimal singular values of matrix B and measures how much the error in the data propagates to the

[1] The upper bound of 1 was considered because of the time-resolution of the data, indeed the minimum spacing among consecutive time points is 1 hour.

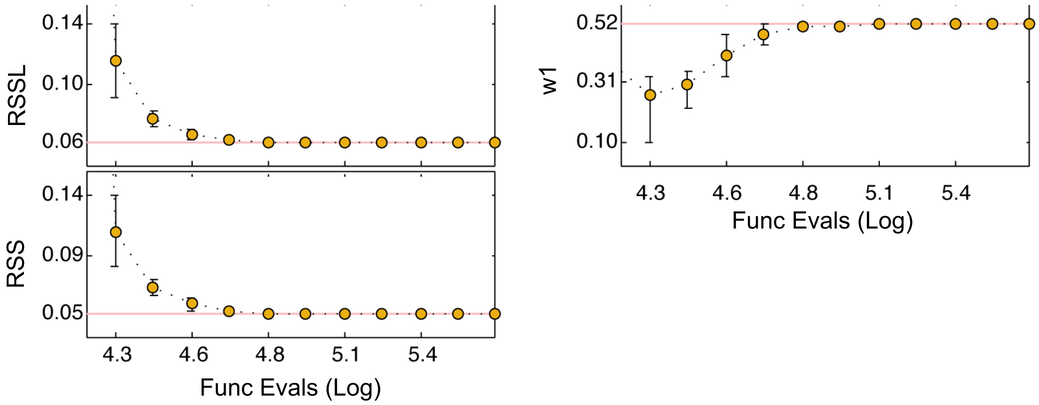

Figure S3. Convergence of the algorithm for the minimization of the reduced RSSL with $k_1 = 256$ and $\lambda = 0.01$. The figure shows the mean of RSSL, RSS and w_1 across 100 minimizations starting from different points as a function of the number of function evals performed by the algorithm. We considered up to $5 \cdot 10^5$ steps in total. Bars represent the first and the fourth quantile of distributions at different steps.

population dynamics. Since over-fitting is characterized by small fluctuations in the data triggering big fluctuations in the estimated optima, the condition number is a good rough estimate of over-fitting. The condition number can be interpreted as a measure of the similarity of states.

To trade-off fit-to-data and model complexity we opted for a Bayesian model selection approach. In the next paragraph we briefly present the Bayesian framework and how we computed the multidimensional integral over model parameters to obtain the posterior distribution over the number of states.

3.2 Bayesian Framework

We here summarize the Bayesian inference approach we employed to determine the most likely number of states describing the data. The aim is to infer the posterior distribution over the number of states. Let M_n denote the model with n states and $y_i(t)$ denote the observed data (normalized to have unitary mean expression and then log-trasformed). Considering normal distributed gene-specific noises over model predictions $\hat{y}_i(t) = \log\left(\sum_j p_j(\mathbf{w}, t)\beta_{ij}\right)$, we have

$$y_i(t) = \hat{y}_i(t) + \epsilon_i \quad \epsilon_i \sim \mathcal{N}\left(0,\ \sigma_i^2\right). \tag{17}$$

Including gene-specific noises σ_i in the parameter set, the model M_n has the $n-1+(n+1)k$ parameters

$$\boldsymbol{\theta} = \{\sigma_1, \boldsymbol{\beta}_1, ..., \sigma_n, \boldsymbol{\beta}_n, \mathbf{w}\} \tag{18}$$

where, as defined above, $\mathbf{w} = (w_{12}, ..., w_{(n-1)n}) \in \mathbb{R}^{n-1}$ is the rate vector containing the $n-1$ transition rates and $\boldsymbol{\beta}_j \in \mathbb{R}^k$ is the expression profile of state j. Taking a flat prior over models ($p(M_n) \propto 1$) the model posterior probability is proportional to the marginal likelihood

$$P(M_n|y_i(t)) = \frac{p(y_i(t)|M_n)\, p(M_n)}{\sum_n p(y_i(t)|M_n)\, p(M_n)} \propto p(y_i(t)|M_n) \tag{19}$$

where the Bayes theorem has been used. The marginal likelihood represents the probability of obtaining our dataset $y_i(t)$ given the model M_n and can be written as an integral over the model parameters

$$p(y_i(t)|M_n) = \int d\boldsymbol{\theta} p(y_i(t)|M_n, \boldsymbol{\theta}) p(\boldsymbol{\theta}|M_n) \tag{20}$$

where $p(\boldsymbol{\theta}|M_n)$ and $p(y_i(t)|M_n, \boldsymbol{\theta})$ are the prior distribution and the likelihood function respectively. To work out this posterior, we used a specific Monte Carlo Markov chain (MCMC) algorithm named 'annealed importance sampling' (AIS) [3]. This algorithm is particularly apt to calculate marginal likelihoods over models and has a very fast convergence rate in comparison with the classical Montecarlo method [4]. In the following we discuss our choices of priors while the AIS algorithm is described in the next paragraph. The priors of the model parameters we employed are:

$$w_{i,i+1} \sim \text{Gamma}(\alpha, \beta) \tag{21}$$
$$\beta_{ij} \sim \text{LogNormal}(\mu, \Sigma) \tag{22}$$
$$\sigma_i \sim \text{InverseGamma}(a_j, b_j) \tag{23}$$

The gamma prior over transition rates penalizes both too low and too high values. Indeed, both very big and very small values of transition rates may lead to states that are never significantly populated across the 32 hours we consider here. Hyperparameters for the gamma distribution were selected to be $\alpha = 2$ and $\beta = 0.15$. This corresponds to assume a prior mean lifetime of states of ~ 6.7 hours. For model expression levels, we expect that their distribution is not very different from the distribution of data expression levels (this leads to $\mu = 0$ and $\Sigma = 0.4$). Finally, concerning the prior over noises, we considered the inverse gamma that best fits the distribution of the standard deviations of gene log-expression across time (this leads to $a = 7.76$ and $b = 1.76$). Doing so, we account for the high level of noise we generally expect to see in microarray measurements.

3.3 Annealed Importance Sampling

In this paragraph we very briefly describe the annealed importance sampling, AIS, algorithm especially focusing on how we set the parameters to improve convergence rates. For further details on the method we refer to [3–5].

For simplicity of notation, in the following we do not explicitly indicate conditioning on the model M_n. Let $0 = \gamma_0 < \gamma_1 < ... < \gamma_M = 1$ be a strictly increasing sequence of real numbers. The annealing bridges the prior and posterior distributions by introducing the $M-1$ additional distributions $p_m(\boldsymbol{\theta}) = p(y_i(t)|\boldsymbol{\theta})^{\gamma_m} p(\boldsymbol{\theta})$. The inverse of γ plays the role of a temperature, and for this reason in the following we refer to γ as "inverse temperature". Let T_m indicate the kernel of the Markov chain having $p_m(\boldsymbol{\theta})$ as equilibrium distribution. Using the AIS method, the marginal likelihood (20) can be written as

$$p\left(y_i(t)\right) \approx \frac{1}{N} \sum_{\alpha=1}^{N} \exp\left\{ \sum_{m=1}^{M} \left(\gamma_m - \gamma_{m-1}\right) \log\left[p\left(y_i(t)|\boldsymbol{\theta}_m^{(\alpha)} \right) \right] \right\} \tag{24}$$

where N is the number of parallel chains, $\boldsymbol{\theta}_1^{(\alpha)}$ is sampled directly from priors whereas $\boldsymbol{\theta}_m^{(\alpha)}$ with $m > 1$ is sampled from $T_{m-1}(\boldsymbol{\theta}|\boldsymbol{\theta}_{m-1}^{(\alpha)})$. We considered $N = 50000$ parallel chains and $M = 10000$ inverse temperatures with the non-linear profiling $\gamma_m = \left(\frac{m}{M}\right)^c$ with $m = 0, ..., M$ and $c = 3$.

Concerning our transition kernel, we considered a Metropolis-within-Gibbs scheme, which provides improved rate of convergence with respect to a naive Metropolis-Hastings approach [6]. This approach also favours the computation of acceptance probabilities for gene-specific parameters since they only depend on parameters relative to that gene. Here we briefly summarize the details of our Gibbs sampling scheme. For each temperature we propose an update for all the component of $\boldsymbol{\theta}$ one by one in this order:

1. for each gene i we firstly propose a transition for its error parameter σ_i and then for its state expressions β_{ij};

2. finally we try a transition for the transition rates.

As proposal distribution for the single component of $\boldsymbol{\theta}$ at inverse temperature γ_m, we considered the probability density function:

$$Q_m(X^\star \mid X) \propto \Theta\left(\max\left\{X - \delta_m, 0\right\} \leq X^\star \leq X + \delta_m\right) \tag{25}$$

where X denotes the starting value of the component of theta we are considering while $X^\star$ denotes the proposed value [2] .To obtain a lower rate of convergence, the domain of the proposal distribution was restricted while the annealed profiling was proceeding, because for higher γ_m (and thus lower temperatures) acceptance rates tend to reduce

[2]As such a proposal distribution is not symmetric the acceptance probability also contains

$$\frac{Q_m(X^\star \mid X)}{Q_m(X \mid X^\star)} = \frac{\delta_m + \min(X, \delta_m)}{\delta_m + \min(X^\star, \delta_m)} \qquad X \geq 0,\ X^\star \geq 0 \tag{26}$$

if the proposal distribution remains the same. In particular δ_m was considered linearly decreasing with γ_m:

$$\delta_m = \delta^{(\max)} + \frac{\gamma_m - \gamma_1}{\gamma_M - \gamma_1}(\delta^{(\min)} - \delta^{(\max)}) \qquad m = 1, ..., M \tag{27}$$

We considered different values of $\delta^{(\max)}$ and $\delta^{(\min)}$ for the three different types of parameter (error parameter, signature parameter and dynamical parameter) and set their values as to yield average acceptance rates of approximately 20-40% for each step of the annealing profile (**Table S1**).

	$\boldsymbol{\delta^{(\min)}}$	$\boldsymbol{\delta^{(\max)}}$
**Error Parameter**	1	2
**Signature Parameter**	1	6
**Dynamical Parameter**	0.1	1

Table S1. Values we used for $\delta^{(\min)}$ and $\delta^{(\max)}$ for the three types of parameters in order to have an acceptance rate of about 20-40%.

We assessed the convergence of our estimate by monitoring the trend of the Bayesian integral and its standard deviation as a function of the number of parallel chains ν ($\nu = 1, ..., N$). We in particular looked at

$$I_\nu = \frac{1}{\nu}\sum_{\alpha=1}^{\nu} \omega^{(\alpha)} \tag{28}$$

$$\Sigma_\nu = \frac{1}{\nu}\sum_{\alpha=1}^{\nu} \left(\omega^{(\alpha)}\right)^2 - \left(\frac{1}{\nu}\sum_{\alpha=1}^{\nu} \omega^{(\alpha)}\right)^2 \tag{29}$$

where

$$\omega^{(\alpha)} = \exp\left\{\sum_{m=1}^{M} (\gamma_m - \gamma_{m-1}) \log\left[p\left(y_i(t)|\boldsymbol{\theta}_m^{(\alpha)}\right)\right]\right\} \tag{30}$$

The plots of I_ν and Σ_ν as function of ν are shown in **Figure S4**, where the convergence of the algorithm is clearly visible. The marginal likelihoods of the model estimated via our AIS scheme are reported in **Table S2** and pictorially represented in Main Text Fig.2c, where we used the normalization

$$P\left(M_n|y_i(t)\right) = \frac{p\left(y_i(t)|M_n\right)}{\sum_{\mu=2}^{7} p\left(y_i(t)|M_\mu\right)}. \tag{31}$$

Monte Carlo standard errors on our estimates in **Table S2** are small, not visible on the scale considered in the plot of Fig.2c in the Main Text. However, we note that while

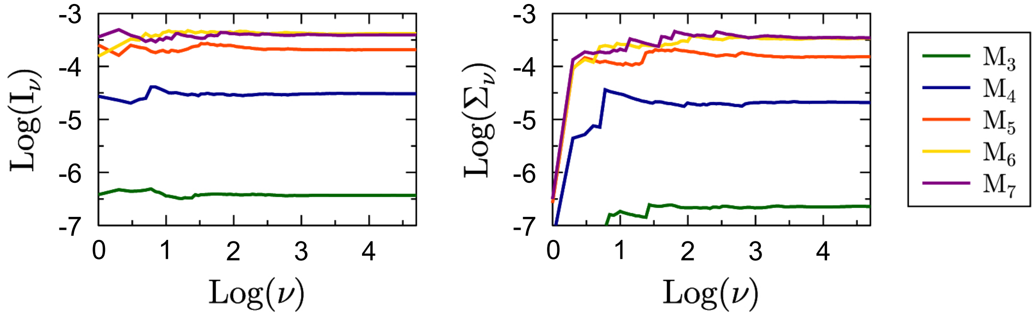

Figure S4. Trend of the mean (left) and standard deviation (left) of weights (independent estimate of the Bayesian integral) calculated over ν parallel chains as function of ν.

Model	Marginal Likelihood
M_2	$(1,612 \pm 0,003)\ 10^{-12}$
M_3	$(3,67 \pm 0,01)\ 10^{-7}$
M_4	$(3,06 \pm 0,01)\ 10^{-5}$
M_5	$(2,069 \pm 0,007)\ 10^{-4}$
M_6	$(4,19 \pm 0,02)\ 10^{-4}$
M_7	$(3,93 \pm 0,02)\ 10^{-4}$

Table S2. Marginal likelihoods estimated via our AIS scheme for models with $2 \div 7$ states with respective standard errors.

the error bars indicate the small magnitude of Monte Carlo error, model selection results depend on several other factors that are not captured in the error bars, including the biological system and data, class of statistical model and prior specification. This analysis was performed on 6 representative genes that capture the dynamics of the system.

References

1. Cicatiello L, Mutarelli M, Grober OMV, Paris O, Ferraro L, et al. (2010) Estrogen receptor alpha controls a gene network in luminal-like breast cancer cells comprising multiple transcription factors and microRNAs. The American journal of pathology 176: 2113–2130.

2. Armond JW, Saha K, Rana AA, Oates CJ, Jaenisch R, et al. (2014) A stochastic model dissects cell states in biological transition processes. Sci Rep 4: 3692.

3. Neal RM (2001) Annealed importance sampling. Statistics and Computing 11: 125–139.

4. Vyshemirsky V, Girolami MA (2008) Bayesian ranking of biochemical system models. Bioinformatics (Oxford, England) 24: 833–839.

5. Friel, Pettitt (2005) Marginal likelihood estimation via power posteriors. J Royal Statistical Society : 1–13.

6. Roberts GO, Rosenthal JS (2006) Harris recurrence of Metropolis-within-Gibbs and trans-dimensional Markov chains. The Annals of Applied Probability 16: 2123–2139.
